# Radiofrequency and Cryoneurolysis in Pain Management: Development, Technique, and Application

**DOI:** 10.1155/prm/7584282

**Published:** 2025-10-16

**Authors:** Paul Elhomsy, Benjamin Reliquet, Gauthier Besson, Rayan Fawaz

**Affiliations:** ^1^Anesthesiology and Critical Care Department, Dijon University Hospital 21000, Dijon, France; ^2^Department of Otolaryngology-Head and Neck Surgery, Dijon University Hospital 21000, Dijon, France; ^3^Department of Physical Medicine and Rehabilitation, Institut Robert-Merle d'Aubigné 94460, Valenton, France; ^4^Department of Neurosurgery, Percy Military Teaching Hospital 92140, Clamart Cedex, France

**Keywords:** chronic pain, cryoneurolysis, guidelines, neurostimulation, pain management, radiofrequency

## Abstract

Radiofrequency and cryoneurolysis are promising options for managing chronic and acute pain. These minimally invasive techniques target nerve structures to alleviate pain in various conditions. This narrative review aims to provide an overview of the historical development, current techniques, applications, and existing evidence for radiofrequency and cryoneurolysis in pain management. While research is ongoing, evidence suggests their effectiveness in treating conditions like postmastectomy pain, phantom limb pain, radicular pain, and trigeminal neuralgia. However, their adoption in clinical guidelines varies, and they carry potential risks. Healthcare professionals should carefully consider the benefits, risks, and current evidence base when making treatment decisions.

## 1. Introduction

Pain, as defined by the International Association for the Study of Pain (IASP), is ‘an unpleasant sensory and emotional experience associated with, or resembling that associated with, actual or potential tissue damage.' Pain has a major impact on patient quality of life. In light of the ongoing opioid crisis, there's a growing push to optimize nonopioid pain management strategies for both chronic and postoperative pain. When nonpharmaceutical treatments or standard drug therapy are insufficient, interventional therapies may represent an effective therapeutic option. These techniques are designed to relieve pain by directly modulating or ablating involved nerve structures and can be used to treat a variety of acute and chronic pain conditions. The aim of this article is to provide a narrative review of interventional radiofrequency and cryoneurolysis therapies for pain management, highlighting their efficiency, safety, and indications, while also discussing the current state of the evidence and their place in clinical guidelines.

## 2. Methods

This article is a narrative review of the literature. To ensure a comprehensive search, we queried the PubMed, Embase, and Cochrane Library databases for studies published between January 2004 and March 2024. The search strategy used the following keywords, alone or in combination: “radiofrequency,” “cryoneurolysis,” “cryoanalgesia,” “pulsed radiofrequency,” “neuropathic pain,” and “pain management.”

Studies were selected for inclusion if they were randomized controlled trials (RCTs), prospective or retrospective cohort studies, systematic reviews, or meta-analyses investigating adult patients experiencing chronic or acute neuropathic pain. The interventions examined were radiofrequency (continuous or pulsed) or cryoneurolysis applied to specific peripheral nerves. Case reports, case series without a control group, preclinical studies, and articles not published in English were excluded. The selection was performed by screening titles and abstracts, followed by a full-text review of potentially relevant articles. Our comprehensive search across the three databases identified approximately 180 records, of which 85 were screened after duplicate removal, and 27 studies were ultimately included in this review.

Given the narrative nature of this review, a formal Preferred Reporting Items for Systematic Reviews and Meta-Analyses (PRISMA) flow diagram was not generated. However, the search was conducted systematically to identify key evidence. The findings are synthesized qualitatively, with a focus on critically appraising the current literature and identifying gaps in evidence.

### 2.1. Radiofrequency

#### 2.1.1. History and Mechanism

Created in the 1950s, the use of a continuous radiofrequency (CRF) generator has gradually evolved. It operates by creating a rapid molecular oscillation through an electromagnetic field, which causes thermal energy and tissue heating.

CRF is the original form of the technique. It applies a continuous electric current to a needle placed near the target nerve, generating temperatures between 60°C and 80°C. This heat results in a well-circumscribed thermal lesion and nerve destruction (neurotomy), thereby alleviating pain [1–4]. CRF affects both myelinated and unmyelinated nerves, including motor neurons, which carries a risk of motor nerve damage and deafferentation pain [4, 5].

Pulsed Radiofrequency (PRF), developed later, PRF applies short-duration electrical pulses (e.g., 20-ms pulses at 2 Hz) interspersed with silent phases. This modality allows for heat dissipation, keeping the tissue temperature below 42°C to avoid irreversible nerve damage [3, 4, 6]. Instead of destruction, PRF is thought to induce neuromodulation by altering the chemical and electrical properties of the nerve signal.

#### 2.1.2. Procedure

Radiofrequency procedures are typically performed on an outpatient basis under local anesthesia. Imaging guidance, most commonly ultrasound or fluoroscopy is used to accurately position the needle at the target nerve. Before delivering the treatment, electrical stimulation is often used to confirm correct needle placement by targeting the nerve that supplies the area of pain and to ensure a safe distance from nontarget motor nerves. Once placement is confirmed, a radiofrequency electrode is inserted through the cannula to create the nerve lesion or apply neuromodulation.

#### 2.1.3. Indications and Efficacy

Radicular Pain: Multiple studies have examined PRF for lumbosacral radiculopathy, with some randomized trials showing efficacy greater than 50% in over 60% of participants, with effects maintained over time [7–13]. However, the evidence remains mixed, as highlighted in French guidelines, with some high-quality studies showing positive results and others showing negative results [14].

Trigeminal Neuralgia: CRF of the Gasserian ganglion has long been an effective but neuro-destructive treatment. PRF has been explored as a less invasive alternative, but studies show divergent results. Some find no significant improvement [15, 16], while others report a decrease in complications compared to CRF [17].

Postherpetic Neuralgia: Several studies, summarized in a meta-analysis by Chang [7], have demonstrated that PRF applied to the dorsal root ganglion (DRG) or intercostal nerve is an effective method for relieving postherpetic neuralgia, proving more effective than medications alone [18–22].

Occipital Neuralgia: Prospective and retrospective studies have shown that PRF of the greater or lesser occipital nerves can provide significant pain relief (over 50% reduction) for at least 3–6 months in a majority of patients who have failed conservative treatments [23–25].

#### 2.1.4. Contraindications and Complications

Adverse effects are generally rare. The most common is transient paresthesias in the treatment area. Rarer complications include infections, nerve injuries, and dysesthesia. Absolute contraindications include increased intracranial pressure and local infection at the puncture site. Relative contraindications include coagulopathy and bacteremia. Due to the risk of electromagnetic interference, specific precautions are recommended for patients with implanted medical devices [26].

### 2.2. Cryoneurolysis

#### 2.2.1. History and Mechanism

Cryoneurolysis employs a cryoprobe to modulate neural activity. It produces reversible axonal injury (axonotmesis) within the target nerve [27]. By lowering the temperature to between −50°C and −70°C using gases like nitrous oxide, ice crystals form within the axon, disrupting its structure and blocking nerve conduction. Crucially, the surrounding connective tissue (endoneurium, perineurium) remains intact, allowing for predictable axonal regeneration at a rate of 1–2 mm per day [28]. This process provides temporary analgesia lasting weeks to months and may reduce the incidence of neuroma formation [29].

#### 2.2.2. Procedure

Similar to radiofrequency, cryoneurolysis is a minimally invasive procedure guided by ultrasound and performed on an outpatient basis. After confirming the target nerve with a diagnostic anesthetic block, a cryoprobe is inserted. Most protocols involve 1 to 3 freeze-thaw cycles, each consisting of 30–120 s of freezing followed by a thawing period. The variability in these protocols across studies is a limitation in the current literature and may contribute to inconsistent results [29–31].

#### 2.2.3. Indications and Efficacy

The primary indication is neuropathic pain, particularly in nerves with minimal or no motor function.

Postmastectomy Pain: A landmark RCT by Ilfeld demonstrated that cryoneurolysis of intercostal nerves (T2-T5) at the time of mastectomy provided superior analgesia for up to 12 months. The study reported a significant reduction in postoperative pain scores and a 98% reduction in cumulative opioid consumption in the first 3 weeks [32].

Postamputation and Phantom Limb Pain: Case studies and a recent multicenter RCT have described ultrasound-guided cryoneurolysis of nerves in the residual limb (e.g., femoral and sciatic) [33]. The initial results are encouraging, showing improvements in pain scores, but more research is needed to standardize protocols [32, 34].

Knee Arthroplasty Pain: To manage prolonged pain after knee arthroplasty, cryoneurolysis of sensory nerves around the knee (e.g., anterior femoral cutaneous nerve and infrapatellar branch of the saphenous nerve) has been studied. A retrospective study by Dasa showed that preoperative cryoneurolysis significantly reduced hospital stays and opioid use [35]. However, this study had a risk of selection bias, and more robust RCTs are needed.

#### 2.2.4. Contraindications and Complications

Contraindications include local infection, coagulopathy, and cold-related disorders such as cold urticaria, Raynaud's syndrome, and cryoglobulinemia. Diabetes is a relative contraindication due to potential for impaired nerve regeneration. Risks include transient neuropathic pain, skin discoloration, or alopecia at the probe site. While motor deficits are a theoretical risk, studies have generally not found impairment of mixed nerve function when performed correctly [27, 30].

## 3. Discussion: Critical Appraisal and Place in Guidelines

While both radiofrequency and cryoneurolysis show significant promise, their current standing in clinical practice is paradoxical; encouraging case-level results and targeted RCTs exist [12, 20, 32, 35] alongside cautious or absent recommendations in major pain guidelines [14, 36]. A critical appraisal of the literature reveals that this gap is not merely due to a lack of evidence but rather to the nature of the evidence itself and the practical barriers to adoption.

A nuanced reading of the evidence suggests that radiofrequency and cryoneurolysis may not be direct competitors but rather complementary tools with distinct therapeutic profiles. Cryoneurolysis, with its mechanism of reversible axonotmesis and strong evidence in the perioperative setting—such as the landmark trial by Ilfeld in postmastectomy pain [32]—is positioning itself as a powerful modality for preventing chronic pain or managing prolonged acute pain. Its temporary, nondestructive nature makes it ideal for contexts where nerve function is expected to recover [27, 28, 30].

Conversely, radiofrequency, particularly CRF, is historically entrenched in treating established chronic pain conditions like facet joint pain and trigeminal neuralgia [15, 37]. The debate should therefore shift from ‘which is better?' to ‘which patient, for which condition, and at what point in their care journey?'

The internal debate within radiofrequency between CRF and PRF perfectly illustrates the broader clinical dilemma in interventional pain medicine. CRF offers proven, superior efficacy in conditions like trigeminal neuralgia [15], but at the cost of neurolysis and the inherent risk of deafferentation pain [4, 38]. PRF, on the other hand, presents a much safer, nondestructive profile by keeping tissue temperatures below the threshold for irreversible damage [3, 5, 6], but its clinical efficacy remains debated and inconsistent across studies [12, 20, 39]. This tension forces a crucial discussion on patient stratification. Should PRF be positioned as a first-line interventional therapy for less severe or higher-risk patients, reserving the more destructive CRF for refractory cases? Or does such a graduated approach merely delay more effective treatment? The lack of a clear consensus on this issue is a significant barrier to standardizing practice [14, 40].

The lag in incorporating these techniques into official guidelines, particularly in France, warrants critical examination. While the primary reason cited is the lack of large-scale, high-quality RCTs [14], this raises a fundamental question: is the traditional RCT the sole appropriate standard for validating operator-dependent, device-based interventions? For procedures where precise patient selection and technical skill are paramount, alternative evidence models—such as pragmatic trials or real-world data from patient registries—should perhaps carry more weight [36, 40] with guideline committees. Waiting for definitive, large-scale RCTs for every specific indication risks depriving patients of promising therapeutic options and stifling innovation in a field still grappling with the opioid crisis (Table 1). As summarized in Table 2, many of the key studies suffer from small sample sizes, single-center designs, or potential risks of bias, which limits the strength of current evidence and underscores the urgent need for more robust trials.

This review has several limitations. First, the heterogeneity of study designs, patient populations, and procedural protocols (particularly in cryoneurolysis freeze–thaw cycles) makes direct comparison between techniques challenging. Second, most of the available evidence consists of small RCTs or observational studies, often single-center, which limits generalizability. Third, the operator-dependent nature of these interventions introduces variability that is difficult to capture in conventional RCTs. Finally, publication bias may have influenced the evidence base, with negative studies potentially underrepresented. These limitations should be considered when interpreting the current evidence and highlight the urgent need for larger, multicenter, and pragmatic trials.

Moving forward, the field requires targeted research priorities to advance evidence-based adoption of these techniques. The most urgent needs include large-scale, multicenter RCTs comparing radiofrequency and cryoneurolysis to standard care across different pain conditions, comprehensive cost-effectiveness studies evaluating long-term healthcare utilization and quality-adjusted life years, standardized protocol development, particularly for cryoneurolysis freeze-thaw cycles, and pragmatic trials that better reflect real-world clinical settings and operator-dependent factors inherent to these procedures.

Finally, the discussion of clinical evidence is incomplete without acknowledging the structural and economic barriers to wider adoption. The significant initial investment in equipment, coupled with the steep learning curve required to master imaging guidance and procedural nuances, currently confines these techniques to specialized centers [26, 38]. This creates a problem of equitable access for patients. Furthermore, without clear endorsement in guidelines and, consequently, robust reimbursement models, the financial incentive for hospitals and clinics to invest in these technologies remains low. A rigorous medico-economic analysis, comparing the one-time cost of a cryoneurolysis procedure to the long-term costs of opioid consumption, repeated consultations, and lost productivity, is essential to demonstrate the value of these interventions to healthcare policymakers [35]. Continued and improved training for healthcare professionals is also a critical component to advance research and accessibility [41].

## 4. Conclusion

Cryoneurolysis and radiofrequency techniques show promise for the treatment of various acute and chronic pain conditions. However, these techniques are not yet widely recognized in official French guidelines due to the need for further research to fully evaluate their efficacy in different indications.

The research presented here demonstrates that both techniques can be used to treat a wide range of pathologies. They should be performed in specialized centers by trained healthcare professionals. These studies also highlight the need for continued research to improve our understanding of these techniques and make them more accessible to patients.

Healthcare professional training is essential to advance research in cryoneurolysis and radiofrequency techniques. Healthcare professionals should be trained in the principles of these techniques, indications, and contraindications, as well as imaging and monitoring techniques.

Training programs can be provided through universities, continuing education courses, or expert-led workshops. It is important that training programs are tailored to the needs of healthcare professionals and are regularly updated based on new scientific evidence.

## Figures and Tables

**Table 1:** Comparison of radiofrequency and cryoneurolysis techniques.

Feature	Radiofrequency (RF)	Cryoneurolysis
Mechanism	CRF: Thermal neurotomy (> 60°C) PRF: Neuromodulation (< 42°C)	Reversible axonotmesis via extreme cold (−50°C to −70°C)
Effect on nerve	CRF: Destructive PRF: Nondestructive	Temporarily disruptive, allows regeneration
Duration of effect	CRF: 6–18 months PRF: Variable, 3–6 months	2–6 months, until nerve regenerates
Primary indications	Radicular pain, trigeminal neuralgia, facet joint pain, postherpetic neuralgia	Postsurgical pain (mastectomy, knee), phantom limb pain, neuralgia in sensory nerves
Key risks	Paresthesia, dysesthesia, nerve injury, infection. Electromagnetic interference with medical devices	Transient neuropathic pain, skin discoloration, motor deficit (rare)
Contraindications	Local infection, increased intracranial pressure, coagulopathy (relative)	Local infection, cold-related disorders (e.g., Raynaud's), coagulopathy (relative)

**Table 2:** Summary of selected key studies.

Study (first author, year)	Condition	Technique	Study design	Key findings and efficacy	Limitations/notes
Van Zundert et al. (2007) [12]	Cervical radicular pain	PRF	Double-blind RCT	PRF group had significantly greater success (pain relief > 50%) at 3 months compared to sham	Relatively small sample size (*n* = 38), single-center design, limited follow-up (3 months). Risk of type II error cannot be excluded
Ilfeld et al. (2023) [32]	Postmastectomy pain	Cryoneurolysis	Multicenter RCT	Superior analgesia up to 12 months; 98% reduction in opioid use in the first 3 weeks	High-quality multicenter RCT with robust methodology, but results are specific to mastectomy patients and may not be generalizable to other surgical contexts
Dasa et al. (2016) [35]	Total knee arthroplasty	Cryoneurolysis	Retrospective cohort	Reduced hospital stay, 45% less opioid use, and improved functional scores	Retrospective, nonrandomized design introduces significant risk of selection bias. Potential confounding factors (rehabilitation protocols, surgical variability) not controlled
Ke et al. (2013) [20]	Thoracic postherpetic neuralgia	PRF	RCT	PRF provided significant and lasting pain relief compared to control group receiving medication only	Single-center study with relatively small sample size. Blinding and allocation concealment not fully described, raising possible risk of bias
Erdine et al. (2007) [15]	Trigeminal neuralgia	PRF versus CRF	Comparative study	CRF was significantly more effective for pain relief than PRF. PRF showed no significant improvement	Comparative study without full randomization. Differences in baseline characteristics between groups may have influenced results

*Note:* References would follow as in the original document, updated as needed.

## Data Availability

Data sharing is not applicable, no new data generated, or the article describes entirely theoretical research.
